# Machine Learning-Based Assessment of Survival and Risk Factors in Non-Alcoholic Fatty Liver Disease-Related Hepatocellular Carcinoma for Optimized Patient Management

**DOI:** 10.3390/cancers16061114

**Published:** 2024-03-10

**Authors:** Miguel Suárez, Sergio Gil-Rojas, Pablo Martínez-Blanco, Ana M. Torres, Antonio Ramón, Pilar Blasco-Segura, Miguel Torralba, Jorge Mateo

**Affiliations:** 1 Gastroenterology Department, Virgen de la Luz Hospital, 16002 Cuenca, Spain; msuarezmatias@sescam.jccm.es (M.S.);; 2 Medical Analysis Expert Group, Institute of Technology, Universidad de Castilla-La Mancha, 16071 Cuenca, Spain; 3 Medical Analysis Expert Group, Instituto de Investigación Sanitaria de Castilla-La Mancha (IDISCAM), 45071 Toledo, Spain; 4 Department of Pharmacy, General University Hospital, 46014 Valencia, Spain; 5 Internal Medicine Unit, University Hospital of Guadalajara, 19002 Guadalajara, Spain; 6 Faculty of Medicine, Universidad de Alcalá de Henares, 28801 Alcalá de Henares, Spain; 7 Translational Research Group in Cellular Immunology (GITIC), Instituto de Investigación Sanitaria de Castilla-La Mancha (IDISCAM), 45071 Toledo, Spain

**Keywords:** non-alcoholic fatty liver disease, hepatocellular carcinoma, NAFLD-related HCC, machine learning, mortality, extreme gradient boosting, alcohol

## Abstract

**Simple Summary:**

Non-alcoholic fatty liver disease (NAFLD) is the most prevalent chronic liver condition globally. The increasing incidence of NAFLD suggests that in the upcoming years, NAFLD-related hepatocellular carcinoma (HCC) is poised to become the leading cause of this type of tumor. The aim of this study is to evaluate the survival rates of these patients and identify the primary risk factors contributing to a less favorable prognosis. To accomplish this, we have employed machine learning techniques. This introduces a novel approach for identifying these factors that can be targeted to enhance the life expectancy of these patients, offering a more personalized and effective management strategy. This enhanced management approach not only aids in the optimization of patient care but also facilitates the delivery of the most effective available treatments.

**Abstract:**

Non-alcoholic fatty liver disease (NAFLD) is the most common chronic liver disease worldwide, with an incidence that is exponentially increasing. Hepatocellular carcinoma (HCC) is the most frequent primary tumor. There is an increasing relationship between these entities due to the potential risk of developing NAFLD-related HCC and the prevalence of NAFLD. There is limited evidence regarding prognostic factors at the diagnosis of HCC. This study compares the prognosis of HCC in patients with NAFLD against other etiologies. It also evaluates the prognostic factors at the diagnosis of these patients. For this purpose, a multicenter retrospective study was conducted involving a total of 191 patients. Out of the total, 29 presented NAFLD-related HCC. The extreme gradient boosting (XGB) method was employed to develop the reference predictive model. Patients with NAFLD-related HCC showed a worse prognosis compared to other potential etiologies of HCC. Among the variables with the worst prognosis, alcohol consumption in NAFLD patients had the greatest weight within the developed predictive model. In comparison with other studied methods, XGB obtained the highest values for the analyzed metrics. In conclusion, patients with NAFLD-related HCC and alcohol consumption, obesity, cirrhosis, and clinically significant portal hypertension (CSPH) exhibited a worse prognosis than other patients. XGB developed a highly efficient predictive model for the assessment of these patients.

## 1. Introduction

Non-alcoholic fatty liver disease (NAFLD) is currently the most prevalent chronic liver disease worldwide. It is known to affect 25–30% of the global population, but is estimated to actually impact around 40% [1]. There are geographical differences within this prevalence [2]. It is defined as the presence of macrovesicular steatosis in ≥ 5% of hepatocytes in the absence of other diseases, such as hepatitis B or C, or excessive alcohol consumption (20 g/day in women and 30 g/day in men) [3]. It is closely correlated with increasing rates of obesity and metabolic comorbidities. Its significance today is such that there are various intercountry projects aimed at addressing this growing issue [4,5]. The definition has also been modified and updated to better characterize patients [6], evolving from the initial definition of NAFLD, transitioning through metabolic-associated fatty liver disease (MAFLD) [7], to the currently proposed definition as metabolic-associated steatotic liver disease (MASLD) [8,9].

The disease progression is erratic, characterized by numerous steps forward and backward between a non-alcoholic fatty liver (NAFL) and non-alcoholic steatohepatitis (NASH) [10]. These changes are influenced by multiple factors driving disease progression, primarily comorbid conditions (diabetes mellitus, insulin resistance, dyslipidemia, obesity, etc.) and lifestyle factors, such as alcohol, physical exercise, coffee consumption, or dietary patterns [11,12]. Additionally, genetic predisposition in each individual plays a significant role, being one of the primary contributors to the heterogeneity among patients [13,14].

It is estimated that only 10% of patients with NAFLD will eventually develop complications stemming from liver disease and cirrhosis [15]. Considering the total number of patients in this situation, this figure is not negligible at all. One of these potential consequences is the development of hepatocellular carcinoma (HCC). HCC is the most frequent primary liver cancer, being the third most common cause of death due to cancer and the sixth in terms of diagnosis [16].

Most HCC cases will develop on a cirrhotic liver irrespective of the etiology. However, when referring to HCC related to NAFLD, this scenario varies. There is a possibility of its development on a non-cirrhotic liver, which poses a significant challenge, particularly concerning its detection [17,18,19].

There is limited published data on the difference in survival between patients with HCC related to NAFLD compared to other causes. The aim of this study is to compare the survival rates among patients with NAFLD-related HCC versus other etiologies. It is also intended to identify prognostic factors that may impact on patient survival. The identification of these factors will facilitate targeted interventions, designed to enhance life expectancy outcomes. For this purpose, machine learning (ML) techniques will be employed. ML presents remarkable superiority by enabling the identification of complex patterns and precise result prediction. These methodologies not only handle large volumes of data efficiently but also have the capability to adapt and learn from the data, unveiling relationships that might go unnoticed using traditional methods [20,21]. The algorithm extreme gradient boosting (XGB) was chosen as the reference method, and it was compared to other systems widely used in the scientific literature [22]. XGB was selected for its fast execution, high scalability, and superior accuracy in results obtained in other medical fields, including hepatology [23,24].

## 2. Materials and Methods

A multicenter retrospective cohort study was conducted at the Virgen de la Luz Hospital in Cuenca and the University Hospital of Guadalajara in Spain. Data collection occurred between January 2008 and December 2022. Inclusion criteria encompassed patients aged 18 and above with a confirmed HCC diagnosis via the presence of an imaging test compatible with its vascular behavior in patients with liver cirrhosis or via liver biopsy for those without cirrhosis or with diagnostic uncertainty [17,25]. Exclusion criteria involved patients diagnosed in a different facility and those lacking available prognostic variables at the time of diagnosis. This study obtained approval from the ethics committee of the University Hospital of Guadalajara. Due to the type of study, an exemption from obtaining informed consent from the patients was granted.

The demographic variables and comorbidities collected were age, sex, date of HCC diagnosis, date of death or censoring date, active alcohol consumption, active smoking, diagnosis of diabetes mellitus (DM) [26] and dyslipidemia (DL) [27], and obesity (considered as a body mass index ≥ 30 kg/m^2^ [28]). The censoring date was considered as the last visit date in the clinic for those patients who were alive. Alcohol consumption was categorized as follows: for patients diagnosed with NAFLD, any amount of alcohol was considered active consumption, taking into account the diagnostic criteria for NAFLD (<20 g/day in women, <30 g/day in men); for the rest of the patients and etiologies, active alcohol consumption was considered as consumption at risk (>20 g/day in women, >30 g/day in men).

Regarding variables related to the diagnosis of HCC, the following were analyzed: etiology (NAFLD vs. any other cause), presence of cirrhosis [29], Child–Pugh score [30], diagnosis within an HCC screening program, Eastern Cooperative Oncology Group (ECOG) stage [31], method of diagnosis (imaging technique or biopsy), model for rnd-stage liver disease (MELD) [32], presence of clinically significant portal hypertension (CSPH) [33], Barcelona Clinic Liver Cancer (BCLC) stage [34], meeting Milan criteria [35], number of lesions, size of the largest lesion (cms), presence of portal thrombosis [36], metastasis, and lymphadenopathy.

Analytical variables collected included the following: neutrophils (cells/mm^3^), lymphocytes (cells/mm^3^), platelets (10^3^/dL), International Normalized Ratio (INR), creatinine (mg/dL), albumin (g/dL), sodium (Na) (mEq/L), bilirubin (mg/dL), Aspartate Aminotransferase (AST) (U/L), Alanine Aminotransferase (ALT) (U/L), and alpha-fetoprotein (AFP) (ng/mL). These variables were collected at the time of diagnosis or within the first month of diagnosis.

To conduct the data analysis, the XGB algorithm was proposed as the reference method. As mentioned earlier, this algorithm was selected for its scalability, execution speed, and excellent results in terms of accuracy. Furthermore, it is a versatile system that allows for parallel computing [37]. Additionally, other ML methods employed in the scientific literature were implemented. These was performed to assess the utility and performance of this system. Those that achieved better performance were support vector machine (SVM) [38], Bayesian linear discriminant analysis (BLDA) [39], decision tree (DT) [40], Gaussian naïve Bayes (GNB) [41], and K-nearest neighbors (KNN) [42]. The resulting models were developed using MATLAB (The MathWorks, Natick, MA, USA; MATLAB R2023a).

To enhance the ML algorithms performance, hyperparameters of each method were adjusted during training utilizing Bayesian techniques in this study. Bayesian optimization, a sequential model-based algorithm, optimized hyperparameter values by leveraging outcomes from previous iterations, reducing the number of model tests, and focusing on parameters likely to improve validation scores [43]. This approach significantly improved the developed model performance. The simulations involved 100 iterations for mean and standard deviation values, reducing noise impact and ensuring statistically valid results [44].

**Figure 1:**
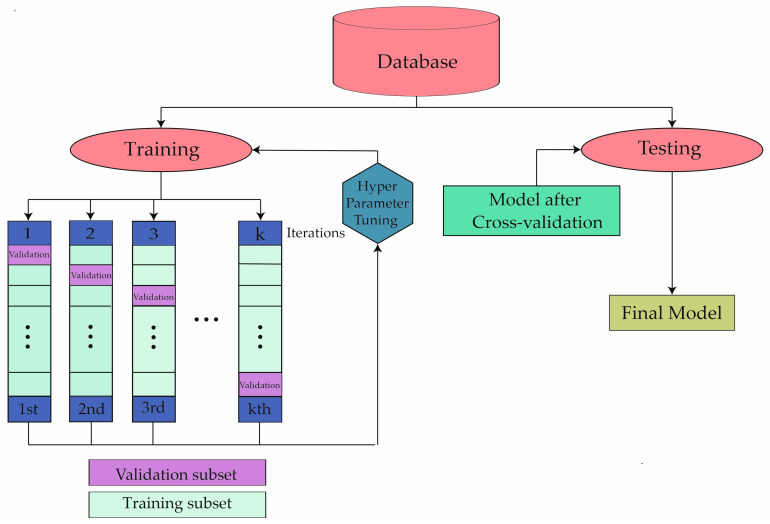
Description of the machine learning methodology development process.

The representation of the steps carried out for the implementation of the ML algorithms is shown in Figure 1. Cross-validation with 5 folds was performed. The analysis was conducted in this manner to prevent overfitting. The developed database was divided into two groups: 70% of it was used in the training phase and the remaining 30% in the testing phase. This approach ensured that patients were not used in both phases simultaneously. After completing this process, the analysis was conducted.

## 3. Results

This section presents the results for the training and validation phases for identifying the main prognostic factors for mortality in patients diagnosed with HCC and NAFLD. It also demonstrates the comparison between (the proposed method) and the rest of the analyzed ML algorithms.

A total of 191 patients were included in the study based on the inclusion and exclusion criteria. Among them, 29 patients developed NAFLD-related HCC, with 24.2% being women. Within this group, low-level alcohol consumption was present in 48.3%. Only 31% of these patients had obesity, and 55.2% were diagnosed with diabetes mellitus (DM). The majority of them (>85%) were incidentally diagnosed outside the HCC screening program, but 41.4% had cirrhosis. Nearly 69% presented with an ECOG score of 0 at diagnosis, and 79.3% had a BCLC score between 0 and A.

In the control group (n = 162), the main causes of HCC were alcohol (38.3%) and hepatitis C (34.6%). Except for patients whose primary cause was alcohol consumption, most of them were either undergoing treatment or had received treatment for their underlying condition causing liver damage. In this group, 61.73% of patients reported abusive alcohol consumption, with DM prevalence at 40.1% and obesity at 31.5%. The percentage of patients with cirrhosis was higher than in the NAFLD group, with 93.2% of patients being cirrhotic. A total of 53% of cases were diagnosed by the screening program. Up to 66.5% of them presented with an ECOG score of 0 at diagnosis, and 42.6% had a BCLC score of 0 or A. More data are available in Table 1.

The average survival within the NAFLD patient group was 9.65 months, while in the rest of etiologies, it approached 12.4 months. The difference between both groups was statistically significant, with a *p*-value of 0.003. Patients with NAFLD-related HCC had portal hypertension (CSPH) diagnosed in 41.38% of cases, compared to 66.66% in the control group. The MELD score was 9 in the NAFLD group, while the other group had a score of 11. Summary of these data is found in Table 2.

**Figure 2:**
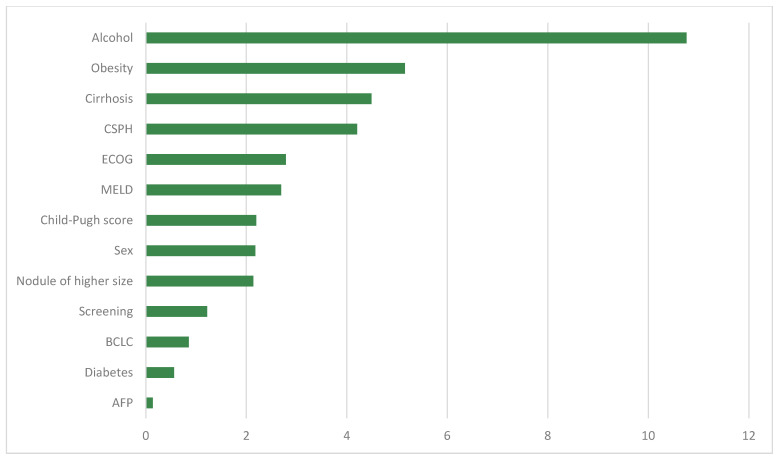
Weight of the most representative variables within the developed predictive model. CSPH: Clinically significant portal hypertension. ECOG: Eastern Cooperative Oncology Group. MELD: model for end-stage liver disease. BCLC: Barcelona Clinic Liver Cancer stage. AFP: alpha-fetoprotein.

In Figure 2, the results of the developed predictive model are presented. Alcohol consumption emerged as the most important variable, followed at some distance by the second variable, obesity. The presence of cirrhosis and the presence of CSPH data were the subsequent variables concerning the mortality of these patients. Both variables showed a similar weight. The prognostic differences among ECOG, MELD, and Child–Pugh stage were not remarkable, as all three presented a similar value. The most significant factor was an advanced ECOG stage. It is noteworthy that alpha-fetoprotein (AFP) levels are insignificant for the prognosis of these patients.

In the next table, the values obtained for various metrics analyzed for the developed models are presented. On one hand, the values for balanced accuracy, recall, specificity, and precision were analyzed. On the other hand, to assess the performance of the methods, area under the curve (AUC), F1 score, Matthews correlation coefficient (MCC), Youden’s dependent index (DYI), and Kappa score were employed. The latter are commonly used methods in the scientific literature for this validation purpose.

As can be observed in Table 3, XGB presents values higher than 94% for balanced accuracy, recall, and specificity, and very close to this value for precision. This implies a significant difference compared to the closest method, KNN, with differences of around 8% for these values. The differences are more substantial for the rest of the algorithms, especially with GNB. In this case, the differences are around 12%. This superiority also translates into the rest of the metrics.

When MCC results are observed, there is a difference of 4.75% between XGB and KNN in favor of the proposed model. This is one of the most reliable statistical indices, yielding high values only when correctly performed across all four categories of the confusion matrix [45]. The differences are significantly higher when comparing the values of F1 score, Kappa, and DYI. Clearly, the differences are also more pronounced for the other proposed algorithms compared to XGB, ranging favorably between 8–12% for XGB. All the aforementioned data are detailed in Table 4.

**Figure 3:**
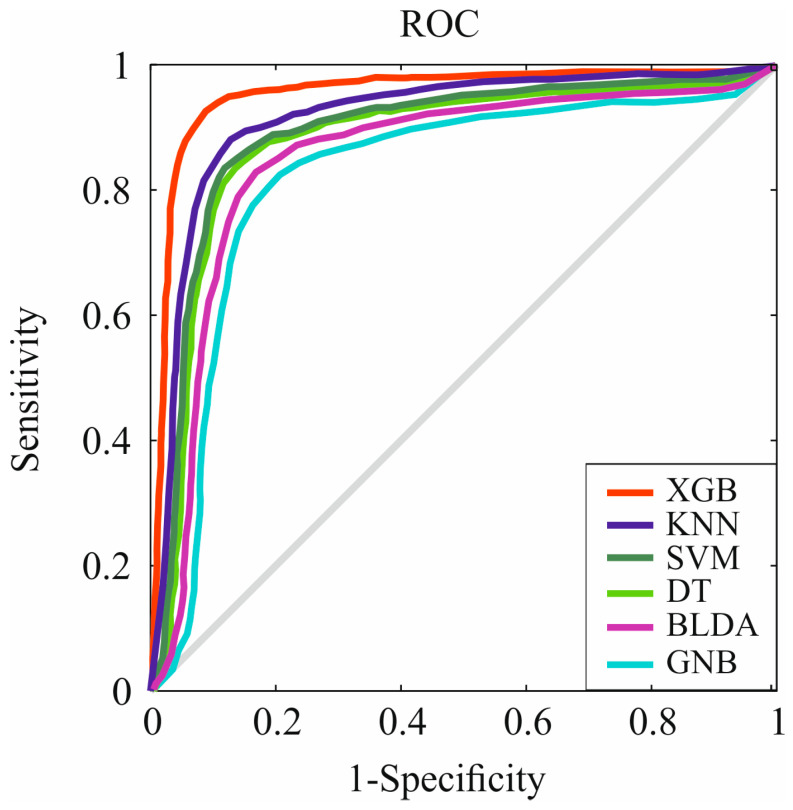
Representation of ROC curves of the analyzed algorithms. XGB: extreme gradient boosting. KNN: K-nearest neighbors. SVM: support vector machine. DT: decision tree. BLDA: Bayesian linear discriminant analysis. GNB: Gaussian naïve Bayes.

Regarding the receiver operating characteristic (ROC) curves, XGB achieves an AUC superior to the rest of the systems. These curves represent sensitivity and specificity for the study’s purpose (Figure 3). The XGB algorithm obtained a value of 0.94, the largest curve among the proposed methods. This higher AUC translates to it being the best method for predicting mortality in patients diagnosed with HCC-NAFLD and for identifying the most influential variables affecting their mortality.

**Figure 4:**
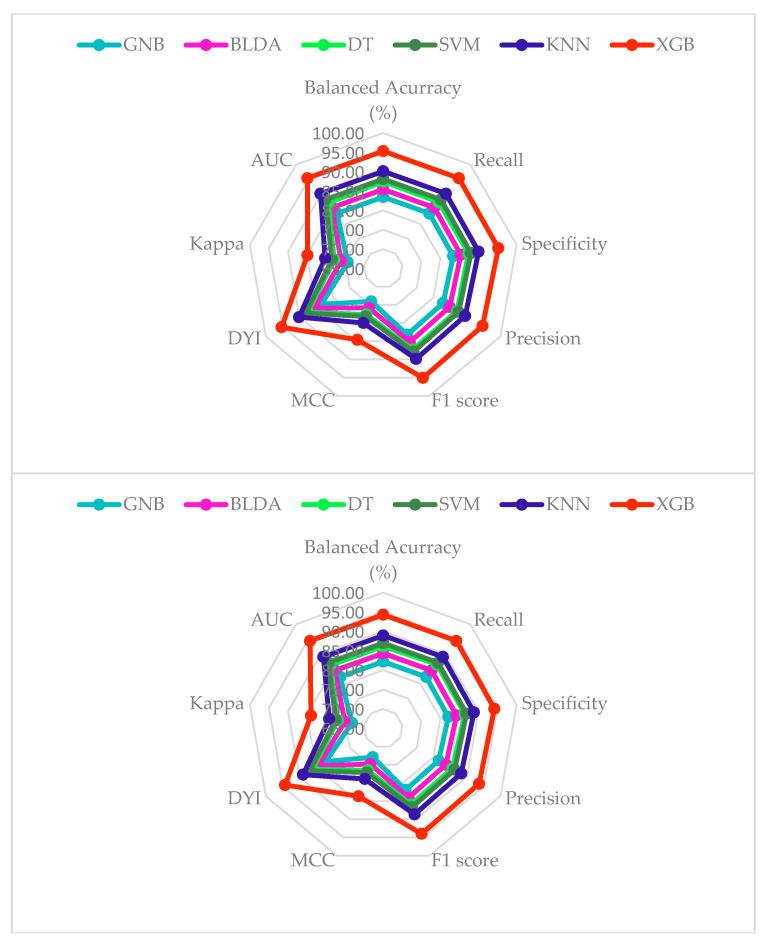
Radar plot comparing all the analyzed methods. The image above is the training phase and the image below is the test phase.

Finally, to depict all this data collectively, a radar plot was created. It showcases the training phase data (above of Figure 4) and the test phase data (below of Figure 4). As observed, the obtained XGB algorithm presents similar data in both phases. This indicates there is no overfitting, implying that the resulting model generates a good predictive model with the capacity for generalizability. A smaller area obtained in this representation implies lower reliability for the study’s objective.

## 4. Discussion

**Figure 5:**
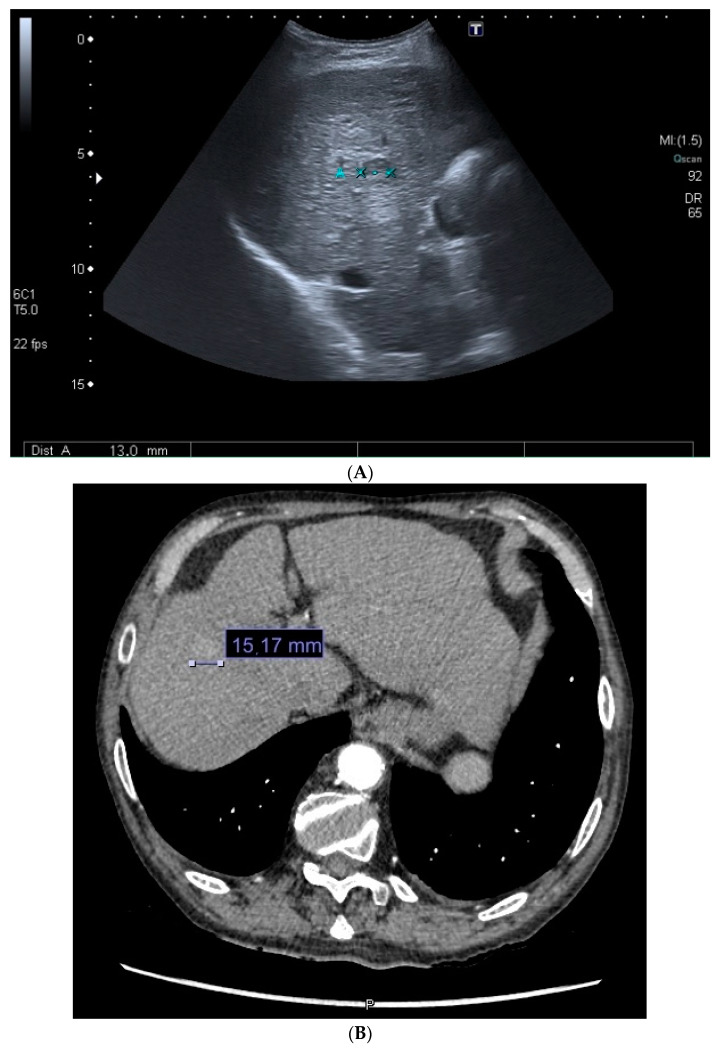

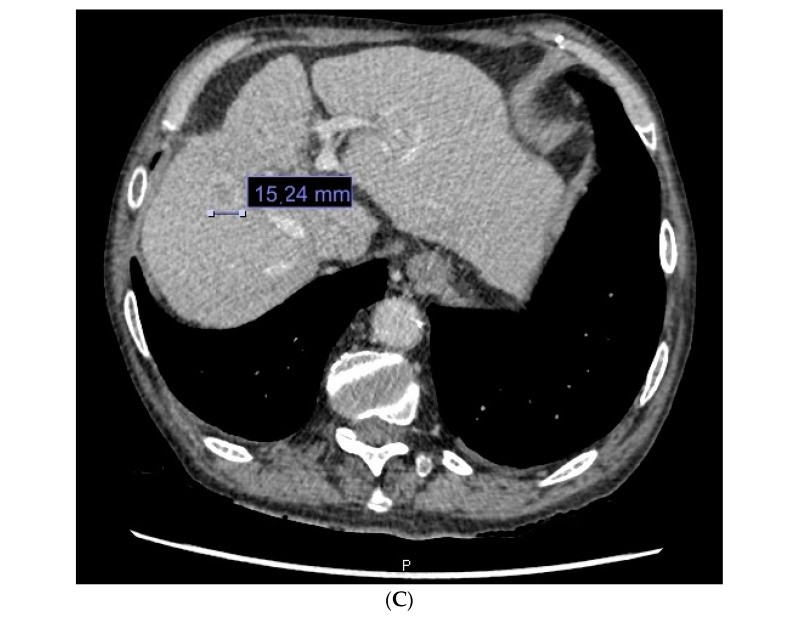
Diagnostic images of HCC. In image (**A**), a hypoechogenic lesion of 13 mm is observed on cirrhotic liver using ultrasound. Image (**B**) displays the arterial phase of the subsequent triphasic CT scan, revealing an image slightly larger than previously described. Finally, image (**C**) shows the venous phase with the characteristic washout, confirming the diagnosis of HCC. HCC: hepatocellular carcinoma. CT: computed tomography.

NAFLD is one of the most relevant chronic diseases today and is clearly on the rise. Since 1990, it is estimated that the prevalence of this disease has risen by 50% [46]. This disease is becoming a significant challenge, especially from the perspective of gastroenterology, particularly hepatology. So much so that different scientific societies and governments of multiple countries are attempting to implement programs and public policies for awareness and to collectively confront this new epidemic [5,47]. The large number of NAFLD patients makes it one of the leading causes of HCC, and in the future, it will be the main one [48,49]. In addition, detecting fibrosis in these patients is complex. This point is crucial because the primary risk factor for this progression is the degree of hepatic fibrosis present, with patients in stages F3-F4 being highlighted, along with the presence of non-alcoholic steatohepatitis (NASH) [50]. To this fact, the possibility of developing NAFL-associated HCC must be added [49]. That is, these patients can develop HCC without significant fibrosis or cirrhosis. The number of patients, its silent nature, and the lack of adequate screening make it impossible for healthcare systems to manage. Moreover, the diagnosis of HCC remains challenging despite the available advances in imaging tests, as depicted in Figure 5.

In line with all the aforementioned information, the trends in HCC etiology are changing. While years ago the primary causes of HCC development were viral hepatitis (chronic hepatitis B and C) and alcohol consumption, NAFLD is now becoming one of the main causes of HCC, already being the leading cause when referring to non-cirrhotic patients [51,52]. Simultaneously, it is also becoming one of the main reasons for liver transplantation. The importance of early detection is crucial since it is a potentially curable tumor. Adequate assessment and monitoring of these patients are essential to detect the disease in time. It is crucial to identify potential risk factors that may contribute to the development of HCC, aiming to act upon them and prevent their occurrence. The list is extensive, including smoking, alcohol consumption, obesity, lifestyle, detection of other underlying liver pathologies, and exposure to certain substances such as aflatoxin [25].

In this study, initially, the differences in mortality between patients with NAFLD-related HCC and other causes are compared. As can be observed, the difference in terms of survival between both groups was statistically significant. Once this was assessed, it was decided to investigate the main prognostic factors at the diagnosis of HCC associated with a poorer prognosis and higher mortality.

It is concluded that the primary risk factor for mortality in these patients is alcohol consumption. Alcohol has been shown to be a potential carcinogen not only at the hepatic level but also in other locations, such as the pancreas or colorectal area, among many others [53]. In this case, alcohol consumption emerges as the worst prognostic factor at the diagnosis of HCC in patients with NAFLD. This can be explained because alcohol acts as an additional incentive for liver damage. Even in low amounts, alcohol consumption enhances the progression of hepatic fibrosis, the degree of inflammation, and the development of HCC [54]. Additionally, although there are no significant histological differences between NASH and alcohol-induced steatohepatitis, alcohol induces characteristic epigenetic changes and alteration in the intestinal microbiome, leading to increased intestinal permeability that may pose a higher risk of HCC [55,56,57].

The second factor with a worse prognosis is obesity. Obesity stands as one of the principal risk factors for the development of NAFLD. These patients also present a higher degree of fibrosis and the possibility of progressing to cirrhosis in proportion to BMI and abdominal circumference. This results from hepatic immune activation, leading to secondary inflammation and fibrosis, heightening the risk of HCC development [58,59]. Additionally, obesity also shows an association and a worse prognosis among patients who consume alcohol. This is explained by the direct relationship between alcohol intake and an increased risk of overweight and obesity [60]. The higher cardio-metabolic risk of these patients is likely an explanation for the worse prognosis.

Other factors associated with a worse prognosis are the presence of cirrhosis and CSPH, both equally significant. It is noteworthy that, despite these results, the Child–Pugh score exhibits lower significance within the predictive model. Unlike the risk factors for HCC development and the published literature, smoking and the presence of DM are not decisive regarding the prognosis of these patients [25]. In fact, being or having been a smoker hardly showed any significance within the predictive model. The low importance of AFP in the prognosis of patients is remarkable. This aligns with the latest scientific evidence published. It is also noteworthy that among the variables related to the tumor, only the largest nodule attains significant value within the predictive model for survival prognosis upon the diagnosis of these patients.

A bibliographic search was conducted on prognostic factors at the diagnosis of HCC focusing on ML techniques, but no results were obtained. Studies conducted previously have focused more on imaging techniques, genomics, and molecular biology [61,62]. For this reason, it was decided to perform the analysis by evaluating various methods. The XGB system demonstrated the best results in all parameters analyzed compared to others. Except for two metrics, the results obtained are around 94%. These figures confirm the utility of XGB for classifying these patients. Moreover, the similarity between the training and test phases shown in the radar plot indicates the absence of overfitting and overtraining. This implies high generalizability, so that when introducing new data, the results obtained are consistent with those obtained at the current time [63]. The method also exhibits high scalability and execution speed, allowing its usefulness in daily clinical practice to assist in decision making.

There are two limitations that need to be addressed. Apart from those inherent in a retrospective study, the primary variable being alcohol consumption raises doubts about whether it was accurately recorded. After analyzing the database, the data were corroborated by at least two researchers from each center, reviewing available medical records of the patients. On the other hand, there might be a question about whether the number of included patients was sufficient to draw these conclusions. To address this, ML techniques can mitigate this issue by optimizing hyperparameters to achieve the highest possible accuracy [64]. These methods more efficiently surpass traditional logistic regressions. The study is replicated 100 times to obtain primary values and standard deviations. This way, statistically significant results are obtained with small samples, also avoiding the potential noise present within [65].

## 5. Conclusions

In conclusion, patients with NAFLD-related HCC exhibit an unfavorable prognosis in terms of survival. Even low alcohol consumption in patients with NAFLD was associated with a poorer prognosis. Obesity, cirrhosis at any stage, and CSPH emerged as additional risk factors conditioning increased mortality at the time of HCC diagnosis in these patients.

XGB proved to be the algorithm that developed a more efficient predictive model in identifying prognostic factors for mortality at the diagnosis of HCC in patients with NAFLD. This model can serve as a valuable tool in the daily management of these patients. Thanks to these results, a more personalized management approach can be offered for these individuals. In addition to the established therapeutic approach, optimizing these conditions may contribute to an improvement in the survival of these patients.

## Figures and Tables

**Table 1:** Summary of descriptive characteristics of included patients. NAFLD: non-alcoholic fatty liver disease. BMI: body mass index. ECOG: Eastern Cooperative Oncology Group. BCLC: Barcelona Clinic Liver Cancer stage.

		Other Etiologies (%)	NAFLD
N		162	29
Sex	Female	18 (11%)	7 (24.2%)
	Male	144 (89%)	22 (75.8%)
Alcohol	None/low risk	62 (38.27%)	None: 15 (51.7%) Low risk: 14 (48.3%)
	Risk	100 (61.73%)	0
Smoker	Never	53 (32.71%)	19 (65.51%)
	Active/ex-smoker	109 (67.28%)	10 (34.48%)
Diabetes mellitus	No	97 (59.87%)	13 (44.82%)
	Yes	65 (40.12%)	16 (55.17%)
Obesity	BMI < 30 kg/m^2^	111 (68.51%)	20 (68.96%)
	≥30 kg/m^2^	51 (34.48%)	9 (31.03%)
Dyslipidemia	No	127 (78.39%)	18 (62.06%)
	Yes	35 (21.6%)	11 (37.93%)
ECOG	0	124 (76.54%)	20 (68.96%)
	1	16 (9.8 7%)	3 (10.34%)
	2	14 (8.64%)	3 (10.34%)
	3	7 (4.32%)	1 (3.44%)
	4	1 (0.61%)	2 (6.89%)
Diagnostic method	Biopsy	54 (33.33%)	16 (55.17%)
	Imaging test	108 (66.66%)	13 (44.87%)
Surveillance	No	76 (46.91%)	25 (86.2%)
	Yes	86 (53.08%)	4 (13.79%)
Cirrhosis	No	11 (6.79%)	12 (41.37%)
	Yes	151 (93.2%)	17 (58.62%)
Etiology	Alcohol	62 (38.27%)	0
	HCV	56 (34.56%)	0
	NAFLD	0	29 (100%)
	Other etiologies	42 (25.92%)	0
CSPH	No	54 (33.33%)	17 (58.62%)
	Yes	108 (66.66%)	12 (41.37%)
Ascites	No	101 (62.34%)	19 (65.51%)
	Yes	61 (37.65%)	10 (34.48%)
Encephalopathy	No	142 (87.65%)	27 (93.1%)
	Yes	20 (12.34%)	2 (6.89%)
Portal thrombosis	No	130 (80.24%)	23 (79.31%)
	Yes	30 (19.75%)	6 (20.69%)
Metastasis	No	146 (90.12%)	21 (72.41%)
	Yes	15 (9.87%)	8 (27.89%)
Lymphadenopathy	No	140 (86.41%)	22 (75.86%)
	Yes	21 (13.58%)	7 (24.13%)
Milan criteria	No	100 (61.73%)	20 (69%)
	Yes	62 (38.27%)	9 (31%)
BCLC	0	9 (5.56%)	1 (3.45%)
	A	60 (37.03%)	11 (37.93%)
	B	27 (16.67%)	2 (6.9%)
	C	48 (29.63%)	10 (34.48%)
	D	18 (11.11%)	5 (17.24%)

**Table 2:** Descriptive values of survival, MELD, and laboratory parameters used. NAFLD: non-alcoholic fatty liver disease. MELD: model for end-stage liver disease. INR: international normalized ratio.

		Mean Value ± Standard Deviation
Survival (months)	Other etiologies	12.4 ± 23.9
	NAFLD	9.65 ± 22.64
MELD	Other etiologies	11
	NAFLD	9
Albumin (g/dL)	Other etiologies	3.70 ± 0.67
	NAFLD	3.58 ± 0.75
INR	Other etiologies	1.27 ± 0.58
	NAFLD	1.20 ± 0.40
Na (mEq/L)	Other etiologies	138.61 ± 3.42
	NAFLD	139.14 ± 3.49
Lymphocytes (cells/mm^3^)	Other etiologies	1436.69 ± 762.29
	NAFLD	1638.21 ± 825.72
Neutrophils (cells/mm^3^)	Other etiologies	3772.69 ± 1999.79
	NAFLD	4663.93 ± 2233.46
Platelets (10^3^/dL)	Other etiologies	140.13 ± 81.60
	NAFLD	178.80 ± 99.15
Creatinine (mg/dL)	Other etiologies	1.02 ± 0.65
	NAFLD	0.95 ± 0.22

**Table 3:** Compilation of the analyzed metrics for all algorithms. SVM: support vector machine. BLDA: Bayesian linear discriminant analysis. DT: decision tree. GNB: Gaussian naïve Bayes. KNN: K-nearest neighbors. XGB: extreme gradient boosting. AUC: area under the curve.

Methods	Accuracy	Recall	Specificity	Precision	AUC
SVM	86.96	87.06	86.85	86.34	0.87
BLDA	84.32	84.42	84.23	83.72	0.84
DT	86.11	86.51	86.41	85.69	0.86
GNB	82.18	82.27	82.08	81.59	0.82
KNN	88.93	89.03	88.82	88.29	0.89
XGB	94.29	94.40	94.18	93.61	0.94

**Table 4:** Summary of metrics collected and analyzed for all methods. SVM: support vector machine. BLDA: Bayesian linear discriminant analysis. DT: decision tree. GNB: Gaussian naïve Bayes. KNN: K-nearest neighbors. XGB: extreme gradient boosting. MCC: Matthews correlation coefficient. DYI: degenerated Youden index.

Methods	MCC	DYI	F1 Score	Kappa
SVM	77.16	86.96	86.70	77.41
BLDA	74.82	84.32	84.07	75.07
DT	76.54	86.11	86.02	76.89
GNB	72.92	82.18	81.93	73.16
KNN	78.91	88.93	88.66	79.17
XGB	83.66	94.29	94.00	83.94

## Data Availability

The datasets used and/or analyzed during the present study are available from the corresponding author on reasonable request.
